# Did inter-hospital transfer reduce mortality in patients with acute myocardial infarction in the real world? A nationwide patient cohort study

**DOI:** 10.1371/journal.pone.0255839

**Published:** 2021-08-05

**Authors:** Mi-Sook Kim, Seong Huan Choi, Jang-Whan Bae, Joongyub Lee, Hyeongsu Kim, Won Kyung Lee

**Affiliations:** 1 Division of Clinical Epidemiology, Medical Research Collaborating Center, Biomedical Research Institution, Seoul National University Hospital, Seoul, Korea; 2 Department of Cardiology, Inha University Hospital, School of Medicine, Inha University, Incheon, Korea; 3 Division of Cardiology, Department of Internal Medicine, Chungbuk National University College of Medicine, Cheongju, Korea; 4 Department of Preventive Medicine, Seoul National University College of Medicine, Seoul, Korea; 5 Department of Preventive Medicine, School of Medicine, Konkuk University, Seoul, Korea; 6 Department of Prevention and Management, Inha University Hospital, School of Medicine, Inha University, Incheon, Korea; Azienda Ospedaliero Universitaria Careggi, ITALY

## Abstract

**Introduction:**

Inter-hospital transfer (IHT) and primary percutaneous coronary intervention (PCI) are preferred over onsite thrombolysis when provided expeditiously. On the other hand, its benefit has not been evaluated in a real-world situation. This study examined the effects of IHT on the short- and long-term mortality in patients with acute myocardial infarction (AMI) and compared the reperfusion treatments and resources between the referring and receiving hospitals.

**Methods:**

Patients newly diagnosed with AMI and admitted to hospital were selected from the national health insurance database from 2004 to 2018. The 30-day and one-year mortality in the transferred and non-transferred patients were estimated and compared using stabilized inverse probability of treatment weighting to account for confounding bias.

**Results:**

Of the 258,291 participants, 10,158 were transferred to one or more hospitals. IHT was more likely to occur to older or more comorbid people, patients in rural areas, and those whose insurance was medical aid. The 30-day and one-year mortality of the non-IHT group was 9.7% and 15.8%, respectively, whereas the figure was 11.4% and 20.5% in the IHT group. After balancing the baseline characteristics, the transferred patients were 1.12 (95% CI: 1.06–1.20) and 1.25 (95% CI: 1.20–1.31) times more likely to die during the subsequent 30 days and one year, respectively, than those treated solely at the presenting hospital. In ST-segment elevation myocardial infarction (STEMI), the hazard ratios of the 30-day and 1-year mortality were 1.14 (95% CI: 0.97–1.35) and 1.31 (95% CI: 1.15–1.49) in the transferred patients after balancing cardiogenic shock and cardiac arrest. On-site thrombolysis was rarely performed in the referring hospitals.

**Conclusion:**

Patients transferred for the treatment of AMI experienced higher short- and long-term mortality. Therefore, onsite thrombolysis and the estimated time delay to PCI should be considered in regional hospitals to reduce mortality with the organization of STEMI treatment networks.

## Introduction

The preference of percutaneous coronary intervention (PCI) over thrombolysis has extended to patients with acute myocardial infarction (AMI) presenting to the hospital without a 24/7 capability of PCI [1, 2]. In 2004, a review of a few randomized controlled trials conducted in Denmark, the Czech Republic, and the US suggested that transferring an ST-segment elevation myocardial infarction (STEMI) patients for primary PCI appears to be superior to onsite fibrinolysis at community hospitals [3]. On the other hand, time delays may hinder inter-hospital transfer (IHT) in routine clinical practice. After 2012, the European Society of Cardiology (ESC) guidelines recommended immediate inter-hospital transfer (IHT) to a PCI center when primary PCI can be made available within the first 120 min of symptom onset [4]. Many cohort studies reported that IHT and primary PCI could be associated with lower mortality than onsite thrombolysis when PCI was provided promptly [5, 6].

It is important to confirm the benefits of primary PCI at the cost of the IHT-related time delay in a real-world situation. The benefits of primary PCI over onsite thrombolysis are conditional on the PCI-related time delay. Moreover, the choice of IHT should be based on the expectation that the maximum delay from symptom onset to primary PCI (wire crossing) is less than 120 minutes [1]. Therefore, the real effect of IHT depends on the traffic condition, the distance between the referring and receiving hospital, regionalization, AMI care network, and IHT protocol. On the other hand, most studies analyzed the regional cohort data, and the study regions were limited to developed countries, such as the US, Australia, Canada, and Italy, where an efficient region-wide system had been built [7, 8].

Therefore, this study examined the effects of IHT on the mortality of patients diagnosed with AMI and evaluated which reperfusion strategy–PCI after transfer vs. onsite thrombolysis–was applied in a real-world setting using national claim data. Furthermore, the characteristics of the transferred hospitals with referring hospitals were compared in terms of healthcare resources and the annual number of PCI.

## Materials and methods

### Data source and study population

This study was performed using the National Health Information Database (NHID), which is the claim database of the National Health Insurance Service (NHIS) in Korea [9]. The NHIS covers the entire population in Korea and is categorized into two insurance types: medical aid and health insurance. The NHID contains the demographic characteristics of the people and information on all health insurance claims, including diagnoses according to the International Classification of Disease 10^th^ revision (ICD-10) and procedures provided in hospitals. The NHID is linked to the national death registration database using the national identification number as a key for the linkage. The NHID was fully anonymized and provided for research purposes. The study protocol was approved by the Institutional Review Board of Inha University Hospital (IRB number: 2021-02-015-0000). It was also approved in a review by the internal committee of NHIS (NHIS study number: NHIS-2021-1-461).

All patients with a principal diagnosis of AMI (ICD-10 code: I21) from 2002 to 2018 were identified, and only the first admission of patients with multiple AMI admissions during the study period was included. The accuracy of the code in the claim data was more than 70%, with the coding of an acute myocardial infarction having a good correlation with the hospital admission discharge records [10].

Patients admitted to a hospital with new-onset AMI and defined as having no claim data on AMI in the preceding two years were enrolled in this study. All participants who were hospitalized through the emergency department (ED) and those older than 18 years were eligible. Patients who were unmatched in the episode database, which were hospitalization episodes reorganized from series of claim data, were excluded.

### Study setting

In Korea, the emergency medical service (EMS) is a single-tiered basic-to-intermediate level system operated by 16 provincial headquarters of the National Fire Department [11]. All EDs were categorized into levels 1, 2, and 3 EDs based on the personnel, equipment, and availability of medical specialties [12]. Level 1 EDs met the highest standard of capacity and capability. Therefore, almost all level 1 EDs can have a PCI team on duty during the day and nighttime. On the other hand, level 2 EDs have variations in the number of cardiologists. Hence, some level 2 EDs could not perform PCI at night and on weekends. PCIs are unavailable at most level 3 EDs. In January 2021, there were 38 level-1 EDs, 128 level-2 EDs and 235 level 3 EDs in Korea [13]. The emergency medical technician (EMT) recommends whether to transport suspicious-AMI patients to the nearest ED of level 1 or 2. An emergency physician decides on immediate thrombolysis vs. transfer for PCI when an AMI-suspicious patient arrives at the hospital where primary PCI was unavailable.

In addition to EDs, thirteen Regional Cardio-cerebrovascular Centers (RCCVCs) have been established to prevent and treat cardiovascular diseases and funded by the Ministry of Health and Welfare [14]. The government planned a two-level system on cardiovascular disease to provide prompt treatment and has operated RCCVC as a level 1 cardiovascular center since 2008; it was preparing to designate a level 2 cardiovascular center.

### Exposure measurements

The primary exposure of this study was IHT, which was defined as a transfer to another emergency room. The IHT should be within one day of visiting an emergency room between acute care facilities to ensure that the visit was not from a long-term facility to an acute care facility [15]. The outcome of interest was the all-cause mortality assessed at 30 days and one year after the onset of AMI.

The covariates were possible confounders, such as age, sex, and underlying diseases: congestive heart failure, peripheral vascular disease, cerebrovascular disease, dementia, chronic pulmonary disease, rheumatic disease, peptic ulcer disease, mild liver disease, diabetes without complication, diabetes with complication, paraplegia/hemiplegia, renal disease, cancer, moderate or severe liver disease, metastatic cancer, and AIDS/HIV [16]. The Charlson comorbidity index (CCI) was calculated from its related conditions in the one-year lookback period before the date of enrollment.

### Subgroup analysis

The subgroups were classified according to the class of AMI: STEMI, non-STEMI (NSTEMI), and unspecified AMI. STEMI and NSTEMI were defined using a principal diagnosis with an ICD-10 four-digit code of I21.0-I21.3 and I21.4, respectively. Unspecified AMI was classified as an ICD-10 code of I21.9 and a three-digit code (I21). The three-digit code of I21, which could not identify the AMI class, accounted for 1.95% of all participants.

Subgroup analysis also adjusted for the severity of AMI: cardiogenic shock and cardiac arrest at the referring hospital. Cardiogenic shock was defined as the condition that required any of the following treatments: inotropes (norepinephrine, dopamine, dobutamine, vasopressin, and milrinone), temporary pacemaker, intra-aortic balloon pump, and extracorporeal membrane oxygenation. Cardiac arrest was defined as the condition when cardiopulmonary resuscitation was provided.

### Statistical analysis

The categorical variables are presented as the frequencies and percentages. The continuous variables are summarized as the mean and standard deviation (SD) or median and inter-quartile range (IQR) in the descriptive analysis. The standardized difference was used to compare the characteristics between the groups who did and did not experience transfer. The survival curves were generated using the Kaplan-Meier estimates and are presented with the log-rank test to compare the survival of the two groups. The Hazard Ratio (HR) and 95% confidence interval (95%CI) were estimated using the proportional hazard model. The stabilized inverse probability of treatment weighting (SIPTW) method was applied to balance the baseline characteristics between the IHT group and the non-IHT group to reduce any confounding bias. The propensity score was estimated using a logistic regression model. The differences in the baseline characteristics between the two groups were evaluated using the standardized differences before and after SIPTW. The HRs with the 95% CIs for the 30-day and one-year mortality were calculated from the proportional hazard model with SIPTW.

The standardized difference was considered significant if its absolute value was >0.1. The other analyses were performed on two sides, and p <0.05 was considered significant. All statistical analyses were performed using the SAS Enterprise Guide (V7.11, SAS Institute, Cary, NC, USA).

## Results

**Fig 1:**
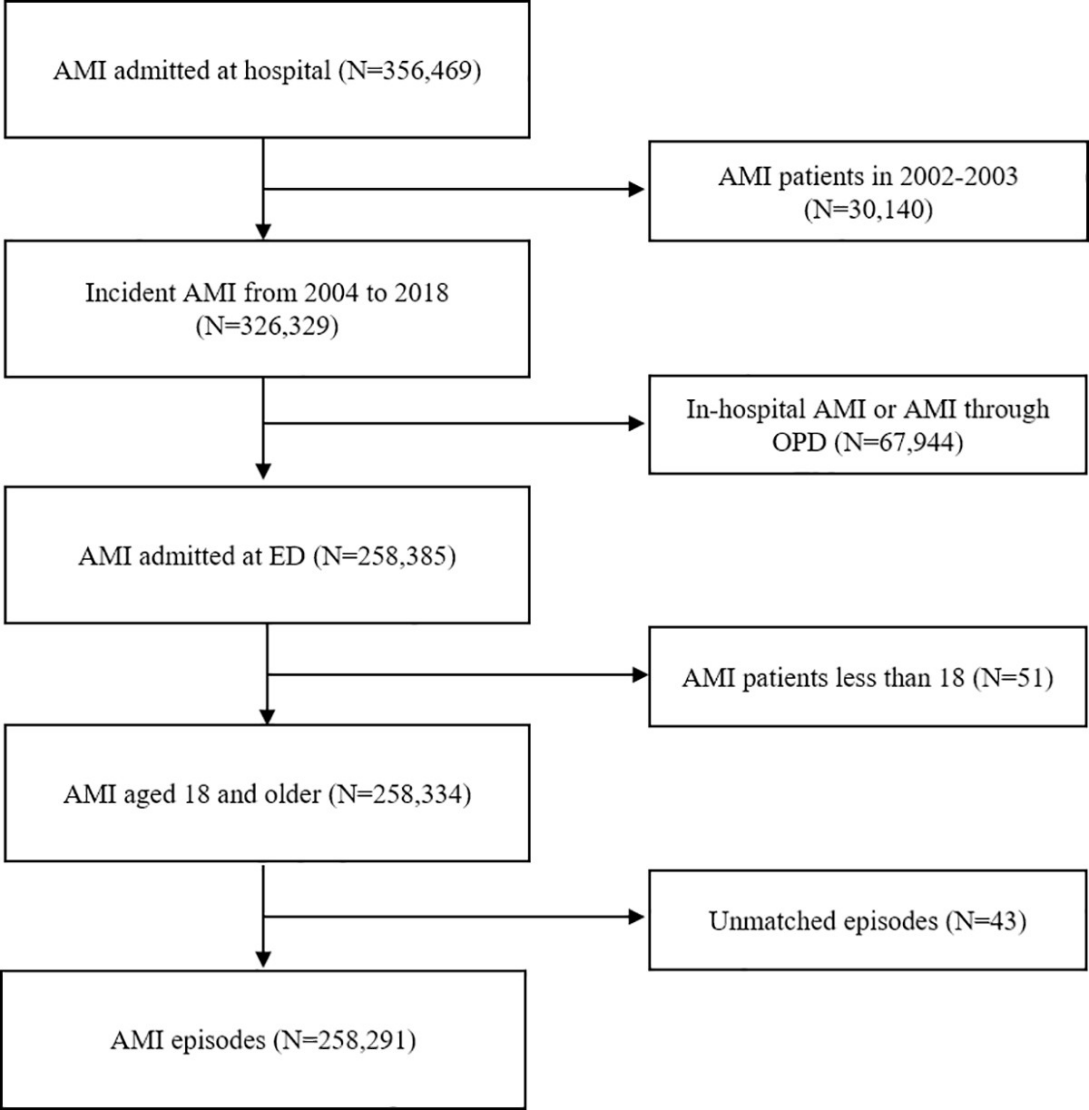
Flowchart of the study inclusion criteria. Abbreviations: AMI, Acute Myocardial Infarction; ED, Emergency Department; OPD, Outpatient Department.

The enrolled participants were 326,329 patients diagnosed with new-onset AMI and admitted to hospital from 2004 to 2018 after excluding 30,140 patients from 2002 to 2003. Of the 326,329 patients, 258,385 patients were hospitalized through the ED, and 51 patients younger than 18 years were excluded. After excluding unmatched patients with episodes in the claim data, 258,291 patients were considered eligible for the final analysis. Fig 1 shows the process of applying the inclusion and exclusion criteria. Among the 258,291 patients analyzed, 76,528 (29.6%), 52,429 (20.3%), and 129,334 (50.1%) was classified into STEMI, non-STEMI, and unspecified AMI, respectively.

Table 1 lists the demographic characteristics according to inter-hospital transfer status. Among the 258,291 patients analyzed, 10,158 patients were transferred between EDs within a day, whereas 248,133 were treated in a hospital. Overall, transfer occurred in older or more comorbid people. The transferred patients had a more underlying disease, such as congestive heart failure, cerebrovascular disease, chronic pulmonary disease, renal disease, and cancer compared to those treated solely at the presenting hospital. No significant difference in the previous PCI and CABG was observed between the IHT and non-IHT groups, but the IHT group was more likely to have prior angina pectoris than the non-IHT group. After applying the SIPTW method, the IHT and non-IHT groups became balanced with the absolute value of the standardized difference <0.1 in the baseline characteristics (Tables 1 and S1).

**Table 1:** Characteristics of the study population.

Characteristics		Non-IHT		IHT		Before SIPTW	After SIPTW
		(N = 248,133)		(N = 10,158)			
		N	%	N	%	Std Diff	Std Diff
Age (mean ± standard deviation)		64.5 ± 13.5		66.6±13.7		0.157	0.023
Sex							
	Male	172,456	69.5	6,702	66.0	-0.075	-0.031
	Female	75,677	30.5	3,456	34.0		
Insurance							
	Medical aid	17,386	7.1	871	8.7	-0.058	-0.016
	Health Insurance	227,788	92.9	9,193	91.4		
Income							
	0 (low)	30,248	12.2	1,509	14.9	0.083	0.021
	1	43,255	17.4	1,716	16.9		
	2	42,345	17.1	1,591	15.7		
	3	51,280	20.7	2,035	20.0		
	4 (high)	81,005	32.7	3,307	32.6		
Region							
	Rural	139,895	56.9	6,869	68.4	-0.240	0.050
	Urban	106,060	43.1	3,172	31.6		
Comorbidity							
	Charlson comorbidity index†	2	0–4	2	1–4	0.260	0.046
	Angina pectoris	56,435	22.7	3,028	29.8	0.161	0.052
	Congestive heart failure	28,307	11.4	2,010	19.8	0.232	0.036
	Peripheral vascular disease	37,955	15.3	1,926	19.0	0.097	0.017
	Cerebrovascular disease	38,053	15.3	2,062	20.3	0.130	0.026
	Dementia	10,540	4.3	678	6.7	0.107	0.027
	Chronic pulmonary disease	72,714	29.3	3,730	36.7	0.158	0.021
	Rheumatic disease	10,671	4.3	516	5.1	0.037	-0.003
	Peptic ulcer disease	63,863	25.7	2,889	28.4	0.061	0.011
	Mild liver disease	52,350	21.1	2,736	26.9	0.137	0.003
	Diabetes without complication	87,331	35.2	4,324	42.6	0.152	0.033
	Diabetes with complication	36,729	14.8	1,856	18.3	0.093	0.025
	Paraplegia /Hemiplegia	4,367	1.8	206	2.0	0.020	0.006
	Renal disease	10,978	4.4	705	6.9	0.109	0.027
	Cancer	14,317	5.8	859	8.5	0.105	0.001
	Moderate or severe liver disease	1,186	0.48	74	0.73	0.032	0.007
	Metastatic cancer	1,638	0.7	100	1.0	0.036	0.008
	AIDS/HIV	133	0.05	4	0.04	-0.007	-0.003
Previous procedure							
	Percutaneous coronary intervention	3,184	1.3	146	1.4	0.013	-0.025
	Coronary artery bypass graft surgery	133	0.05	14	0.14	0.027	0.014

†Median and Interquartile range.

IHT, Inter-hospital transfer; SIPTW, Stabilized Inverse Probability of Treatment Weighting; Std Diff, Standardized difference.

In the IHT group, 1,162 patients had expired within 30 days after hospital admission with a higher crude and weighted mortality rate than the non-IHT group (Table 2). The crude 30-day mortality in non-IHT and IHT groups was 9.7% and 11.4%, respectively. The one-year mortality in non-IHT and IHT groups was 15.8% and 20.5%, respectively. The difference between the two groups was reduced after applying SIPTW for 30-day and one-year mortality.

**Table 2:** Cumulative incidence of the 30-day and one-year mortality in acute myocardial infarction patients according to the transfer status.

Outcomes	Incidence	Non-IHT	IHT
30-day mortality	Number of event	24,154	1,162
	Crude cumulative incidence	9.7%	11.4%
	Weighted cumulative incidence	10.3%	11.6%
1-year mortality	Number of event	39,161	2,087
	Crude cumulative incidence	15.8%	20.5%
	Weighted cumulative incidence	16.7%	20.7%

IHT, Inter-hospital transfer.

**Fig 2:**
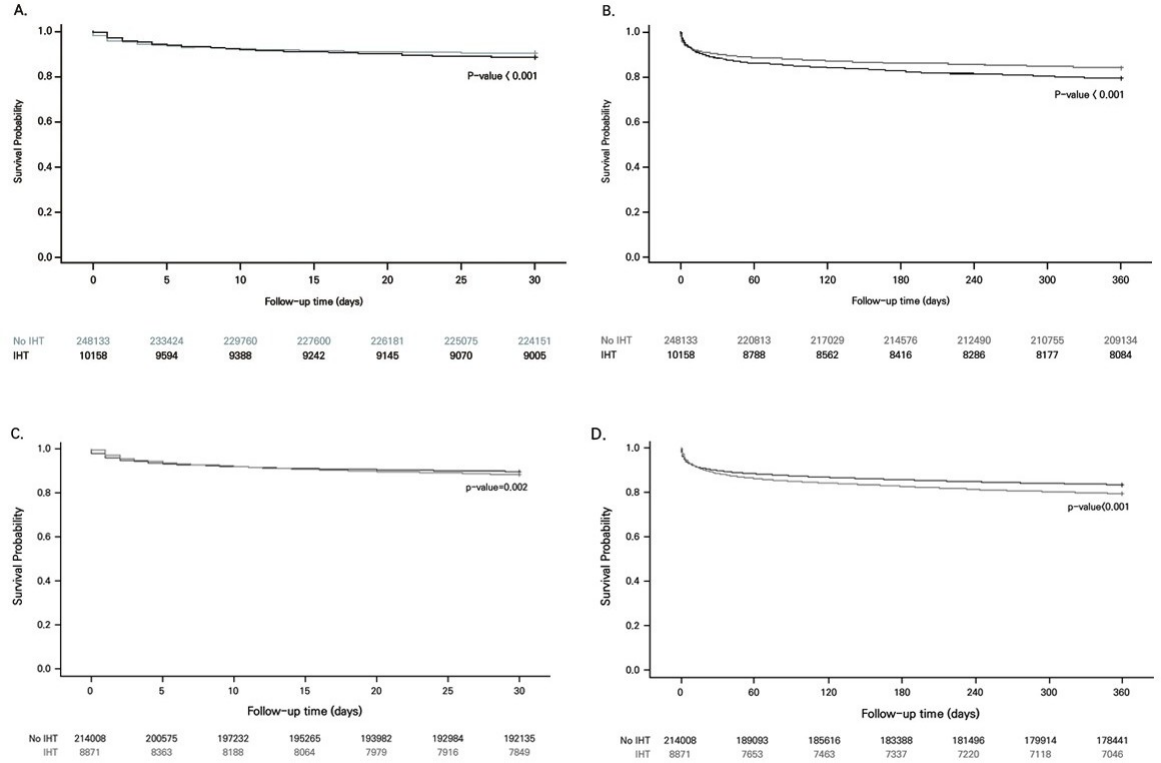
Survival curve of the 30-day and 1-year mortality in acute myocardial infarction patients grouped by inter-hospital transfer status before and after the standardized inverse probability of treatment weighting. A: Survival curve of the 30-day mortality, B: Survival curve of the 1-year mortality, C: Adjusted survival curve of the 30-day mortality, D: Adjusted survival curve of the 1-year mortality. Abbreviations: IHT, Inter-hospital transfer.

**Fig 3:**
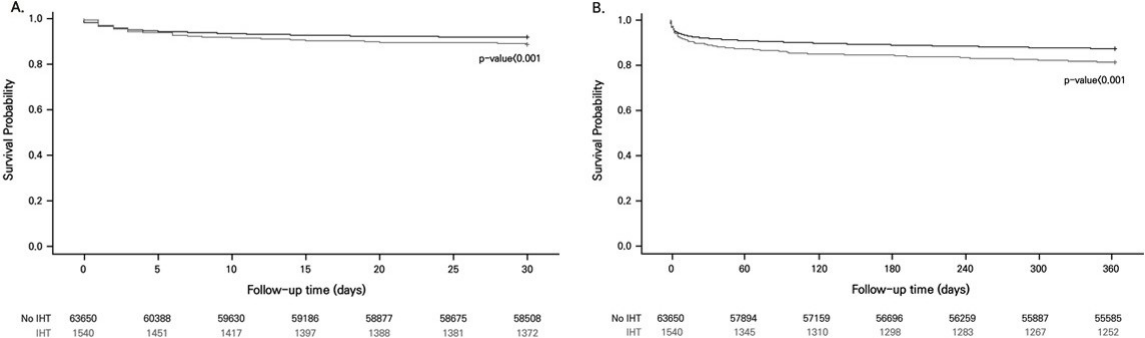
Survival curve of the 30-day and 1-year mortality in ST-segment elevation myocardial infarction patients grouped according to the inter-hospital transfer status after the standardized inverse probability of treatment weighting. A: Adjusted survival curve of the 30-day mortality, B: Adjusted survival curve of the 1-year mortality. Abbreviations: IHT, Inter-hospital transfer.

Fig 2 shows the difference in the 30-day and one-year mortality of AMI patients. The IHT group had significantly higher mortality at 30 days and one year after AMI onset (p-value < .001). The difference remained significant in the one-year mortality (p-value < .001) and the 30-day mortality (p-value = .002) when SIPTW was used. Regarding STEMI, the IHT group was also more likely to have higher one-year mortality (p-value < .001) and 30-day mortality (p-value < .001) (Fig 3).

The transferred patients showed higher mortality at 30-days than those treated at the presenting hospital. The higher mortality of the IHT group compared to the non-IHT group was more prominent at one year after admission (Table 3). The transferred patients were 12.3% (95%CI: 1.055–1.196) and 25.3% (95%CI: 1.196–1.313) more likely to die within 30 days and one year. For STEMI, the weighted HRs were 1.348 (95%CI: 1.157–1.570) and 1.481 (95%CI: 1.317–1.665); IHT had a higher risk of mortality in patients with STEMI.

**Table 3:** Crude and weighted hazard ratios of transfer on the short- and long-term mortality after balancing comorbidity and demographic characteristics.

	Mortality	Group	Crude HR (95% CI)	P-value	*Weighted HR (95% CI)	P-value
Total AMI	30-day	IHT	1.174 (1.106–1.245)	< .001	1.123 (1.055–1.196)	< .001
	1-year	IHT	1.320 (1.263–1.379)	< .001	1.253 (1.196–1.313)	< .001
STEMI	30-day	IHT	1.400 (1.210–1.620)	< .001	1.348 (1.157–1.570)	< .001
	1-year	IHT	1.532 (1.368–1.715)	< .001	1.481 (1.317–1.665)	< .001
NSTEMI	30-day	IHT	1.268 (1.102–1.460)	< .001	1.175 (0.985–1.403)	.074
	1-year	IHT	1.340 (1.220–1.472)	< .001	1.243 (1.104–1.399)	< .001
Unspecified AMI	30-day	IHT	1.077 (1.001–1.158)	.046	0.988 (0.917–1.064)	.744
	1-year	IHT	1.203 (1.138–1.272)	< .001	1.109 (1.048–1.174)	< .001

IHT, Inter-hospital transfer; HR Hazard Ratio; CI Confidence Interval.

*Model was balanced for demographic characteristics (age, sex, insurance type, income level, region), underlying disease, Charlson comorbidity index, and previous PCI and CABG using Weighted for Standardized Inverse Probability of Treatment.

The effect of IHT on mortality also depended on the severity of AMI (S2 Table). The IHT group tended to have higher mortality than the non-IHT group when the patients did not have cardiogenic shock or cardiac arrest at the referring hospital. The transferred patients without cardiogenic shock were 29.7% (95%CI: 1.112–1.511) and 44.6% (95%CI: 1.331–1.571) more likely to die within 30 days and one year, respectively. For those without cardiac arrest, the weighted HRs were 1.358 (95%CI: 1.248–1.477) and 1.409 (95%CI: 1.332–1.490). On the other hand, IHT did not increase or decrease the risk of mortality among those who were in shock, whereas the IHT group was less likely to die among those who were in cardiac arrest.

When the severity of AMI was balanced, the effects of IHT on mortality diminished but persisted (Table 4). For STEMI and NSTEMI, the transferred patients were 30.8% (95%CI: 1.153–1.485) and 18.4% (95%CI: 1.049–1.336) more likely to die within one year, respectively. The weighed HRs were 1.142 (95%CI: 0.965–1.351) and 1.098 (95%CI: 0.915–1.318) in evaluating 30-day mortality for STEMI and NSTEMI, which did not reach statistical significance. On the other hand, IHF in unspecified AMI was associated with lower mortality (HR: 0.860, 95%CI: 0.793–0.932) that did not persist in the one-year follow-up.

**Table 4:** Crude and weighted hazard ratios of transfer on the short- and long-term mortality according to the class of acute myocardial infarction.

	Covariates	30-day mortality		1-year mortality	
		*Weighted HR (95% CI)	P-value	*Weighted HR (95% CI)	P-value
STEMI	Crude	1.400 (1.210–1.620)	< .001	1.532 (1.368–1.715)	< .001
	Model 1	1.348 (1.157–1.570)	< .001	1.481 (1.317–1.665)	< .001
	Model 2	1.247 (1.065–1.462)	.006	1.394 (1.236–1.573)	< .001
	Model 3	1.195 (1.015–1.407)	.033	1.349 (1.192–1.526)	< .001
	Model 4	1.142 (0.965–1.351)	.123	1.308 (1.153–1.485)	< .001
NSTEMI	Crude	1.268 (1.102–1.460)	< .001	1.340 (1.220–1.472)	< .001
	Model 1	1.175 (0.985–1.403)	< .001	1.243 (1.104–1.399)	< .001
	Model 2	1.103 (0.921–1.320)	.287	1.215 (1.080–1.368)	.001
	Model 3	1.120 (0.935–1.342)	.218	1.188 (1.053–1.340)	.005
	Model 4	1.098 (0.915–1.318)	.314	1.184 (1.049–1.336)	.006
Unspecified AMI	Crude	1.077 (1.001–1.158)	.046	1.203 (1.138–1.272)	< .001
	Model 1	0.988 (0.917–1.064)	.744	1.109 (1.048–1.174)	< .001
	Model 2	0.964 (0.895–1.039)	.341	1.109 (1.048–1.174)	< .001
	Model 3	0.878 (0.811–0.951)	.001	1.016 (0.957–1.079)	.595
	Model 4	0.860 (0.793–0.932)	< .001	1.006 (0.947–1.069)	.838

*Weighted for Standardized Inverse Probability of Treatment

Model 1: demographic characteristics (age, sex, insurance type, income level, region), underlying disease, Charlson comorbidity index, and previous PCI and CABG.

Model 2: Model 1+ cardiogenic shock.

Model 3: Model 1+ cardiac arrest.

Model 4: Model 1+ cardiogenic shock + cardiac arrest.

Table 5 lists the capacities and resources of the referring hospitals and receiving hospitals. The median number of doctors and specialists working in the receiving hospitals were 221 and 116, respectively, whereas there were 51 and 45 in the referring hospitals. The median number of beds in the general ward and ICU were 600 and 39, respectively, in the receiving hospitals, which were higher than those in the referring hospitals. Differences in the annual number of operations and procedures were also observed in the referring and receiving hospitals. The median numbers of PCI were 152 in the referring hospital, whereas it was 842 in the receiving hospital. As a result, transferred patients are more likely to be treated by primary PCI in the receiving hospital. Among the transferred patients, 56.1% received PCI in the receiving hospitals, whereas 5.9% received PCI in the referring hospitals (Table 6). CABG (Coronary Artery Bypass Graft) was provided to 6.0% of transferred patients in the receiving hospitals but not in the referring hospitals. In the subgroup of STEMI, NSTEMI, and unspecified AMI, most PCI or CABD was provided in the receiving hospitals. The proportion of the revascularization therapy in STEMI, NSTEMI, and unspecified AMI was 85.8%, 70.2%, and 61.9%, respectively.

**Table 5:** Resources and volume of care in the referring and receiving hospitals where the transferred patients with acute myocardial infarction visited.

		Referring hospital		Receiving hospital		*P-value*
		Median	IQR	Median	IQR	
Healthcare resource	Doctor	51	22–204	221	69–347	< .001
	Specialist	45	21–110	116	53–170	< .001
	Bed	363	228–589	600	393–780	< .001
	ICU	23	14–41	39	25–53	< .001
Annual procedure	CABG	0	0–7	30	9–105	< .001
	PCI	152	0–479	842	499–1320	< .001

IQR, Inter Quartile Range; ICU, Intensive Care Unit; PCI, Percutaneous Coronary Intervention; CABG, Coronary Artery Bypass Graft.

**Table 6:** Procedures provided for patients with acute myocardial infarction before and after inter-hospital transfer.

		Referring hospital		Receiving hospital		*P-value*
		N	%	N	%	
AMI	CABG	0	0.0	612	6.0	< .001
(N = 10,222)	PCI	599	5.9	5,739	56.1	< .001
	Thrombolysis	5	0.05	14	0.14	.039
STEMI	CABG	0	0.0	83	4.7	< .001
(N = 1,749)	PCI	176	10.1	1,236	70.6	< .001
	Thrombolysis	0	0.0	3	0.3	.250
NSTEMI	CABG	0	0.0	175	6.4	< .001
(N = 2,749)	PCI	68	2.5	1,688	61.4	< .001
	Thrombolysis	0	0.0	0	0.0	
Unspecified AMI	CABG	0	0.0	354	6.2	< .001
(N = 5,715)	PCI	355	6.2	2,814	49.2	< .001
	Thrombolysis	5	0.1	11	0.2	.134

PCI, Percutaneous Coronary Intervention; CABG, Coronary Artery Bypass Graft.

## Discussion

This study showed that the transferred patients have higher mortality at 30 days and one year after the onset of AMI, where the national transfer protocol was not established. Elderly and patients with other comorbidities were more likely to be transferred to another ED. These patients with STEMI or NSTEMI were less likely to survive in the short and long-term, even after balancing the baseline characteristics and severity of AMI. Moreover, the transferred patients rarely received thrombolysis before inter-hospital transfer and were less likely to be treated with reperfusion treatment promptly.

Primary PCI is the preferred reperfusion treatment in STEMI patients [17]. In addition to RCTs performed previously, observational studies also documented the potential for a similar or favorable outcome of the primary PCI group despite longer delay than the onsite thrombolysis group. Several cohort studies had a narrower spectrum of patients than the whole population in claim data for the following reasons: 1) STEMI patients with >12hr or missing data on time from symptom onset to hospital arrival were excluded [6]; 2) those who underwent rescue PCI or early PCI after thrombolysis (4.2%) and with incomplete data (13.8%) were excluded [7]; 3) the eligibility criteria was 3–6 hours from symptom onset to hospital arrival and those who refused IHT were excluded [8]; 4) those admitted at hospitals with less than 15 patients in the transfer were excluded [18]. Therefore, previous studies suggested that the effectiveness of primary PCI could be different in an unselected population within various institutions [7].

The various patterns of the imbalance in the baseline characteristics between the IHT group and non-IHT group among previous studies were also noticeable. Some studies reported that patients transferred with AMI were younger and had fewer comorbidities than those treated solely at the presenting hospital [19, 20]; other studies had similar baseline characteristics [7, 8]. In other studies, however, the transferred patients had a more pre-hospital cardiac arrest, a higher rate of cardiovascular risk factors, and Killip classes 3 or 4 on presentation [5, 6]. These differences could act as confounding factors when the outcomes between the IHT group vs. the non-IHT group were evaluated in the observational studies. Therefore, Gale et al. suggested that the confounding factors should be mitigated as best as possible using statistical methods, such as propensity score analysis and inverse probability of treatment weighting [2].

The findings of this study were in the same direction as the previous research using the multicenter registry of high-volume hospitals [21]. However, the results of the one-year mortality of STEMI patients by IHT did not reach statistical significance. Moreover, it was difficult to compare the findings of the present study directly with those of the previous study because different exclusion criteria had been applied: a symptom-to-door time > 12 hours, a door-to-balloon time > six hours, and missing symptom-to-door time. Although the difference in mortality between the transfer group vs. direct-arrival group had marginal significance due to the small sample size, they reported a difference in mortality, which may have clinical relevance: in-hospital mortality in the transfer group vs. direct-arrival group (5.1% vs. 3.9%), one-month mortality (5.9% vs. 5.1%), and 12-month mortality (8.2% vs. 6.6%). The lack of a well-established network and regionalization for AMI care and the resulting time delay might have contributed to the higher mortality in the transferred patients. The protocol of the Korean emergency medical service (EMS) is not based on the facility or personnel for the treatment of AMI. Nevertheless, suspicious-AMI patients are supposed to be transported to level-1 or level-2 EDs. RCCVCs were not incorporated into the transportation guidelines because of the absence of a level 2 cardiovascular center and its conflict with the level of ED. Furthermore, direct transfer from the referring hospital to the catheterization laboratory has been unavailable. Therefore, it may also contribute to reperfusion delay for primary PCI and worsen the mortality in transferred patients [22].

A recent study showed that only 29.3% of patients had a door-to-balloon time of fewer than 120 minutes in Korea, and the median length of stay in the referring hospital, inter-hospital transport time, and the door-to-balloon time of the receiving hospital was 50, 32, and 55 minutes, respectively [23]. In the real world, the transfer of STEMI patients might not follow the guidelines that primary PCI is the preferred reperfusion treatment in STEMI patients if it can be performed within 120 min of the first medical contact; otherwise, fibrinolysis should be considered [1]. A previous study on the largest registry in the US also reported similar findings that only 42.6% of transfer patients could be treated with PCI within 120 minutes of the first door-to-balloon time when the estimated drive time exceeded 30 minutes [24]. This study suggested that fibrinolysis is rarely used despite the low likelihood of achieving timely PCI [25]. Only 0.05% of patients received thrombolysis in the referring hospital, where they spent a median of 50 minutes. The number appears to be much lower than 34.3% of eligible patients in the US who received pre-transfer fibrinolysis with an estimated drive time of 30 to 120 minutes [24]. Furthermore, the referring hospitals spent as much as 50 minutes diagnosing AMI and preparing IHT, whereas it took 55 minutes for physicians to start primary PCI in the receiving hospitals. In the guidelines, each target time for the diagnosis of AMI using twelve-lead ECG and fibrinolysis is 10 minutes [1]. Therefore, lack of a proper on-site thrombolysis and IHT protocol could also contribute to the higher mortality of the transferred patients compared to those treated solely at the presenting hospital.

Among the 10,158 patients transferred to another hospital, 479 (4.7%) patients had a cardiac arrest at the referring hospital, and 841 (8.3%) patients had a cardiac arrest at any of the referring or the receiving hospitals within one day of hospital admission. The number was similar to the 7.9% of patients treated solely at the presenting hospital. The proportion of cardiac arrests in the IHT and non-IHT groups was not significantly different (standardized difference = .016), and cardiac arrest might not be associated with IHT. On the other hand, the influence on IHT might be different if cardiac arrest increases or decreases IHT. If the patients with cardiac arrest were less likely to be resuscitated and transferred, the severity of the IHT group would be lower than that of the non-IHT group. Therefore, it would move the effect of IHT on mortality toward the null or the reverse causality. This could occur when only the patients who were successfully resuscitated could be transferred, which IHT may have different effects on mortality according to cardiac arrest in S2 Table. Lastly, but less likely, it would worsen the mortality of the IHT group if those with cardiac arrest were more likely to be transferred. Therefore, the results were suggested with those additionally adjusted for the severity of AMI using cardiac arrest and cardiogenic shock.

After adjusting for the severity, IHT was still related to a higher risk of mortality in STEMI but was less prominent in NSTEMI patients. This disparity is in line with the pathophysiological mechanism of these two disease entities [26]. STEMI results from plaque rupture of an atheroma resulting in thrombus formation and the total occlusion of any of the epicardial arteries. Therefore, the clinical manifestation is more rapid and time-dependent. The consequences of time delay due to IHT would ultimately escalate the patient’s mortality in the long term. On the other hand, NSTEMI is the cardiac ischemia caused by an imbalance of oxygen demand and supply to the cardiac muscle. The extent of epicardial luminal narrowing vary from moderate narrowing to pending plaque rupture and, in some cases, total occlusion with collateral circulation formation. For NSTEMI patients, the clinical presentation is diverse compared to STEMI, ranging from non-symptomatic patients to cardiogenic shock and cardiac arrest. Therefore, IHT-related time delay may not compromise the short-term mortality because of these varying clinical presentations and wide spectrum coronary pathophysiology. On the other hand, both STEMI and NSTEMI patients were at higher risk of one-year mortality adjusted for the severity, reinforcing the argument that the symptom to balloon time is closely associated with the myocardial infarct size.

The proportion of the treated patients diagnosed with AMI was low, approximately 70%. This was consistent with the previous study on the claim database that the invasive treatments, including PCI and CABG, provided to patients with AMI was 70.2% [25]. The proportion of invasive treatment provided to patients depended on the type of AMI; it was highest in STEMI and lowest in unspecified AMI. When the proportion of the treatment provided is evaluated, it may be helpful to consider the accuracy of the diagnostic codes in the claim database. In the validation study, the accuracy was 71.4% according to World Health Organization criteria and 73.1% according to the European Society of Cardiology/American College of Cardiology (ESC/ACC) criteria [10]; it was lower in those who were 50 years or older. Furthermore, the in-hospital mortality, the general condition of the patients, and the contraindication of PCI and thrombolysis may also help in the decision for invasive treatment. The proportion of comorbidity in patients newly diagnosed with AMI was 15.5% with stroke, 5.9% with cancer, and 0.7% with metastatic cancer.

This study is subject to the usual limitations of claims data. First, this study could be biased despite evaluating the causal relationship between IHT and short- and long-term mortality. IHT occurs in the clinical field where human factors, referring hospital factors, and clinical factors interact. Unobserved variables may also act as covariates between IHT and the outcomes, even though IPTW depends on the causal diagram and mimics a randomized controlled trial in an observational study. Furthermore, clinical information was limited because the claim database did not collect clinical laboratory and vital signs. Therefore, elaborate and exquisite analysis using clinical information will be needed in a future study. Second, the type of AMI could not be well distinguished in the claim database using ICD-10 codes. Indeed, a large proportion was classified into unspecified AMI and may occur when the clinician does not distinguish the type of AMI and administers the diagnostic code in the electronic medical records. On the other hand, subgroup analysis showed that IHT was related to higher mortality in STEMI. Third, an evaluation of the severity was limited because it did not contain clinical information, such as laboratory findings and vital signs; however, the severity of AMI was considered in this study using the medication and procedure for cardiogenic shock and cardiac arrest. On the other hand, it has national representativeness and is free of selection bias that has limited the generalization of clinical trials or registries. Moreover, comparability was maximized by SIPTW, which is accepted as an optimal statistical approach to derive an unbiased estimate of the treatment effect when patients are not assigned randomly to an observational study.

## Conclusion

The results of this nationwide retrospective observational study suggest that the IHT of AMI patients in places where regionalization was not well established could be associated with a higher mortality. Harmonized AMI care network, IHT protocol integrated with the estimated time delay will reduce the mortality of AMI patients. Furthermore, the quality should be monitored by the performance indicators and mortality using claim data.

## Supporting information

S1 Table Differences in the characteristics of the study population before and after the inverse probability of treatment weighting. (DOCX)

S2 Table Crude and weighted hazard ratios of transfer on the short- and long-term mortality according to the severity of acute myocardial infarction. (DOCX)

## References

[1] Ibanez B, James S, Agewall S, Antunes MJ, Bucciarelli-Ducci C, Bueno H, et al. 2017 ESC Guidelines for the management of acute myocardial infarction in patients presenting with ST-segment elevation. European Heart Journal. 2018;39(2):119–77. doi: 10.1093/eurheartj/ehx393 28886621

[2] Gale CP, Van Laar M, Hamm C. Acute myocardial infarction and inter-hospital transfer. Heart. 2015;101(13):998–9. doi: 10.1136/heartjnl-2015-307508 25800998

[3] Waters RE, Singh KP, Roe MT, Lotfi M, Sketch MH, Mahaffey KW, et al. Rationale and strategies for implementing community-based transfer protocols for primary percutaneous coronary intervention for acute ST-segment elevation myocardial infarction. Journal of the American College of Cardiology. 2004;43(12):2153–9. doi: 10.1016/j.jacc.2003.12.057 15193673

[4] Steg PG, James SK, Atar D, Badano LP, Lundqvist CB, Borger MA, et al. ESC Guidelines for the management of acute myocardial infarction in patients presenting with ST-segment elevation. European Heart Journal. 2012;33(20):2569–619. doi: 10.1093/eurheartj/ehs215 22922416

[5] Mueller S, Zheng J, Orav EJ, Schnipper JL. Inter-hospital transfer and patient outcomes: A retrospective cohort study. BMJ Quality and Safety. 2019;28(11):E1-E. doi: 10.1136/bmjqs-2018-008087 30257883 PMC11128274

[6] Kawecki D, Gierlotka M, Morawiec B, Hawranek M, Tajstra M, Skrzypek M, et al. Direct Admission Versus Interhospital Transfer for Primary Percutaneous Coronary Intervention in ST-Segment Elevation Myocardial Infarction. JACC: Cardiovascular Interventions. 2017;10(5):438–47. doi: 10.1016/j.jcin.2016.11.028 28216215

[7] Manari A, Ortolani P, Guastaroba P, Casella G, Vignali L, Varani E, et al. Clinical impact of an inter-hospital transfer strategy in patients with ST-elevation myocardial infarction undergoing primary angioplasty: The Emilia-Romagna ST-segment elevation acute myocardial infarction network. European Heart Journal. 2008;29(15):1834–42. doi: 10.1093/eurheartj/ehn323 18617475

[8] Zhao X, Yang X, Gao C, Chu Y, Yang L, Tian L, et al. Improved survival of patients with ST-segment elevation myocardial infarction 3–6 hours after symptom onset is associated with inter-hospital transfer for primary percutaneous coronary intervention (PCI) at a large regional st-segment elevation myocardial. Medical Science Monitor. 2017;23:1055–63. doi: 10.12659/msm.902466 28240997 PMC5341906

[9] Cheol Seong S, Kim YY, Khang YH, Heon Park J, Kang HJ, Lee H, et al. Data Resource Profile: The National Health Information Database of the National Health Insurance Service in South Korea. Int J Epidemiol. 2017;46(3):799–800. Epub 2016/10/31. doi: 10.1093/ije/dyw253 ; PubMed Central PMCID: PMC5837262. 27794523 PMC5837262

[10] Kimm H, Yun JE, Lee SH, Jang Y, Jee SH. Validity of the diagnosis of acute myocardial infarction in Korean National Medical Health Insurance claims data: The Korean Heart Study. Korean Circulation Journal. 2012;42(1):10–5. doi: 10.4070/kcj.2012.42.1.10 22363378 PMC3283749

[11] Lee J, Lee W, Lee YJ, Sim H, Lee WK. Effectiveness of bystander cardiopulmonary resuscitation in improving the survival and neurological recovery of patients with out-of-hospital cardiac arrest: A nationwide patient cohort study. PLoS One. 2020;15(12):e0243757. Epub 2020/12/17. doi: 10.1371/journal.pone.0243757 ; PubMed Central PMCID: PMC7744051. 33326454 PMC7744051

[12] Choi YH, Lee YJ, Shin SD, Song KJ, Lee KW, Lee EJ, et al. The impact of recommended percutaneous coronary intervention care on hospital outcomes for interhospital-transferred STEMI patients. American Journal of Emergency Medicine. 2017;35(1):7–12. doi: 10.1016/j.ajem.2016.09.024 27771225

[13] National_Emergency_Medical_Center. National Emergency Medical Center, South Korea 2021 [cited 2021 1-Mar]. Available from: https://www.e-gen.or.kr/nemc/main.do.

[14] Kim A, Yoon SJ, Kim YA, Kim EJ. The burden of acute myocardial infarction after a regional cardiovascular center project in Korea. Int J Qual Health Care. 2015;27(5):349–55. Epub 2015/08/15. doi: 10.1093/intqhc/mzv064 . 26271544

[15] Kim HS, Kang DR, Kim I, Lee K, Jo H, Koh SB. Comparison between urban and rural mortality in patients with acute myocardial infarction: A nationwide longitudinal cohort study in South Korea. BMJ Open. 2020;10(4):1–6. doi: 10.1136/bmjopen-2019-035501 32273319 PMC7245421

[16] Quan H, Sundararajan V, Halfon P, Fong A, Burnand B, Luthi JC, et al. Coding algorithms for defining comorbidities in ICD-9-CM and ICD-10 administrative data. Med Care. 2005;43(11):1130–9. Epub 2005/10/15. doi: 10.1097/01.mlr.0000182534.19832.83 . 16224307

[17] Bhatt DL. Percutaneous Coronary Intervention in 2018. JAMA. 2018;319(20):2127–8. Epub 2018/05/26. doi: 10.1001/jama.2018.5281 . 29800163

[18] Barbash IJ, Zhang H, Angus DC, Reis SE, Chang CCH, Pike FR, et al. Differences in Hospital Risk-standardized Mortality Rates for Acute Myocardial Infarction When Assessed Using Transferred and Nontransferred Patients. Medical Care. 2017;55(5):476–82. doi: 10.1097/MLR.0000000000000691 28002203 PMC5391291

[19] Burstein B, Bibas L, Rayner-Hartley E, Jentzer JC, van Diepen S, Goldfarb M. National Interhospital Transfer for Patients With Acute Cardiovascular Conditions. CJC Open. 2020;2(6):539–46. doi: 10.1016/j.cjco.2020.07.003 33305214 PMC7711006

[20] Ranasinghe I, Barzi F, Brieger D, Gallagher M. Long-term mortality following interhospital transfer for acute myocardial infarction. Heart. 2015;101(13):1032–40. doi: 10.1136/heartjnl-2014-306966 25736049

[21] Kim BW, Cha KS, Park MJ, Choi JH, Yun EY, Park JS, et al. The impact of transferring patients with st-segment elevation myocardial infarction to percutaneous coronary intervention-capable hospitals on clinical outcomes. Cardiology Journal. 2016;23(3):289–95. doi: 10.5603/CJ.a2016.0003 26779970

[22] Anderson LL, French WJ, Peng SA, Vora AN, Henry TD, Roe MT, et al. Direct Transfer From the Referring Hospitals to the Catheterization Laboratory to Minimize Reperfusion Delays for Primary Percutaneous Coronary Intervention: Insights From the National Cardiovascular Data Registry. Circ Cardiovasc Interv. 2015;8(9):e002477. Epub 2015/09/05. doi: 10.1161/CIRCINTERVENTIONS.114.002477 . 26338881

[23] Park JH, Ahn KO, Shin SD, Cha WC, Ryoo HW, Ro YS, et al. The first-door-to-balloon time delay in STEMI patients undergoing interhospital transfer. American Journal of Emergency Medicine. 2016;34(5):767–71. doi: 10.1016/j.ajem.2015.12.058 26926589

[24] Vora AN, Holmes DN, Rokos I, Roe MT, Granger CB, French WJ, et al. Fibrinolysis use among patients requiring interhospital transfer for st-segment elevation myocardial infarction care a report from the us national cardiovascular data registry. JAMA Internal Medicine. 2015;175(2):207–15. doi: 10.1001/jamainternmed.2014.6573 25485876

[25] Larson DM, McKavanagh P, Henry TD, Cantor WJ. Reperfusion Options for ST Elevation Myocardial Infarction Patients with Expected Delays to Percutaneous Coronary Intervention. Interventional Cardiology Clinics. 2016;5(4):439–50. doi: 10.1016/j.iccl.2016.06.004 28581994

[26] Hong JS, Kang HC. Sex Differences in the Treatment and Outcome of Korean Patients With Acute Myocardial Infarction Using the Korean National Health Insurance Claims Database. Medicine (Baltimore). 2015;94(35):e1401. Epub 2015/09/04. doi: 10.1097/MD.0000000000001401 ; PubMed Central PMCID: PMC4616509. 26334894 PMC4616509

