# Supplementary material for: Did inter-hospital transfer reduce mortality in patients with acute myocardial infarction in the real world? A nationwide patient cohort study
Source: PLoS One. 2021 Aug 5;16(8):e0255839. doi: 10.1371/journal.pone.0255839 (PMC8341481; doi:10.1371/journal.pone.0255839)
Supplement: S1 Table — (DOCX) [file pone.0255839.s001.docx]

**S1 Table. Characteristics of the study population after Standardized Inverse Probability of Treatment Weighting**

| Characteristics | | Non-IHT  (N=214,008) | | IHT  (N=8,871) | | After SIPTW |
| --- | --- | --- | --- | --- | --- | --- |
|  |  | N | % | N | % | Std Diff |
| Age (mean ± standard deviation) | | 65.1 ± 13.3 | | 65.4±13.8 | | 0.023 |
| Sex | |  |  |  |  |  |
|  | Male | 145,503 | 68.0 | 5,902 | 66.5 | -0.031 |
|  | Female | 68,505 | 32.0 | 2,969 | 33.5 |  |
| Insurance | |  |  |  |  |  |
|  | Medical aid | 15,808 | 7.4 | 692 | 7.8 | -0.016 |
|  | Health Insurance | 198,200 | 92.6 | 8,179 | 92.2 |  |
| Income | |  |  |  |  |  |
|  | 0 (low) | 24,808 | 11.6 | 1,061 | 12.0 | 0.021 |
|  | 1 | 37,669 | 17.6 | 1,582 | 17.8 |  |
|  | 2 | 36,244 | 16.9 | 1,466 | 16.5 |  |
|  | 3 | 44,858 | 21.0 | 1,811 | 20.4 |  |
|  | 4 (high) | 70,430 | 32.9 | 2,951 | 33.3 |  |
| Region | |  |  |  |  |  |
|  | Rural | 133,691 | 62.5 | 5,326 | 60.0 | 0.050 |
|  | Urban | 80,317 | 37.5 | 3,545 | 40.0 |  |
| Comorbidity | |  |  |  |  |  |
|  | Charlson comorbidity index† | 2 | 0-4 | 2 | 0-4 | 0.046 |
|  | Coronary artery disease | 50,697 | 23.7 | 2,302 | 25.9 | 0.052 |
|  | Congestive heart failure | 23,956 | 11.2 | 1,095 | 12.3 | 0.036 |
|  | Peripheral vascular disease | 33,607 | 15.7 | 1,447 | 16.3 | 0.017 |
|  | Cerebrovascular disease | 34,461 | 16.1 | 1,515 | 17.1 | 0.026 |
|  | Dementia | 9,187 | 4.3 | 431 | 4.9 | 0.027 |
|  | Chronic pulmonary disease | 65,876 | 30.8 | 2,819 | 31.8 | 0.021 |
|  | Rheumatic disease | 9,425 | 4.4 | 386 | 4.4 | -0.003 |
|  | Peptic ulcer disease | 56,709 | 26.5 | 2,395 | 27.0 | 0.011 |
|  | Mild liver disease | 45,996 | 21.5 | 1,918 | 21.6 | 0.003 |
|  | Diabetes without complication | 78,418 | 36.6 | 3,394 | 38.3 | 0.033 |
|  | Diabetes with complication | 33,109 | 15.5 | 1,455 | 16.4 | 0.025 |
|  | Paraplegia /Hemiplegia | 3,946 | 1.8 | 169 | 1.9 | 0.006 |
|  | Renal disease | 9,524 | 4.5 | 446 | 5.0 | 0.027 |
|  | Cancer | 12,666 | 5.9 | 526 | 5.9 | 0.001 |
|  | Moderate or severe liver disease | 1,046 | 0.5 | 48 | 0.5 | 0.007 |
|  | Metastatic cancer | 1,457 | 0.7 | 66 | 0.7 | 0.008 |
|  | AIDS/HIV | 78 | 0.04 | 3 | 0.03 | -0.003 |
| Previous procedure | |  |  |  |  |  |
|  | Percutaneous coronary intervention | 2,757 | 1.3 | 91 | 1.0 | -0.025 |
|  | Coronary artery bypass graft surgery | 121 | 0.06 | 8 | 0.09 | 0.014 |

†Median and Interquartile range

IHT, Inter-hospital transfer; SIPTW, Standardized Inverse Probability of Treatment Weighting; Std Diff, Standardized difference
