# Supplementary material for: Did inter-hospital transfer reduce mortality in patients with acute myocardial infarction in the real world? A nationwide patient cohort study
Source: PLoS One. 2021 Aug 5;16(8):e0255839. doi: 10.1371/journal.pone.0255839 (PMC8341481; doi:10.1371/journal.pone.0255839)
Supplement: S2 Table — (DOCX) [file pone.0255839.s002.docx]

**S2 Table. Crude and weighted hazard ratios of transfer on the short- and long-term mortality according to the severity of acute myocardial infarction**

| Condition | | *Weighted HR (95% CI) | | |
| --- | --- | --- | --- | --- |
|  |  | 30-day mortality |  | 1-year mortality |
| Shock | No | 1.297 (1.112-1.511) |  | 1.446 (1.331-1.571) |
|  | Yes | 0.977 (0.913-1.047) |  | 1.069 (1.010-1.132) |
|  |  |  |  |  |
| Cardiac arrest | No | 1.358 (1.248-1.477) |  | 1.409 (1.332-1.490) |
|  | Yes | 0.731 (0.666-0.802) |  | 0.784 (0.719-0.855) |
